# A Critical Role of Glutamine and Asparagine γ-Nitrogen in Nucleotide Biosynthesis in Cancer Cells Hijacked by an Oncogenic Virus

**DOI:** 10.1128/mBio.01179-17

**Published:** 2017-08-15

**Authors:** Ying Zhu, Tingting Li, Suzane Ramos da Silva, Jae-Jin Lee, Chun Lu, Hyungjin Eoh, Jae U. Jung, Shou-Jiang Gao

**Affiliations:** a Department of Molecular Microbiology and Immunology, Keck School of Medicine, University of Southern California, Los Angeles, California, USA; b Key Laboratory of Gene Engineering of the Ministry of Education, State Key Laboratory of Biocontrol, School of Life Sciences, Sun Yat-sen University, Guangzhou, Guangdong, People's Republic of China; c Department of Microbiology and Immunology, Nanjing Medical University, Nanjing, Jiangsu, People's Republic of China; d Laboratory of Human Virology and Oncology, Shantou University Medical College, Shantou, Guangdong, People's Republic of China; Virginia Polytechnic Institute and State University

**Keywords:** γ-nitrogen, asparagine, cancer, glutamine, KSHV

## Abstract

While glutamine is a nonessential amino acid that can be synthesized from glucose, some cancer cells primarily depend on glutamine for their growth, proliferation, and survival. Numerous types of cancer also depend on asparagine for cell proliferation. The underlying mechanisms of the glutamine and asparagine requirement in cancer cells in different contexts remain unclear. In this study, we show that the oncogenic virus Kaposi’s sarcoma-associated herpesvirus (KSHV) accelerates the glutamine metabolism of glucose-independent proliferation of cancer cells by upregulating the expression of numerous critical enzymes, including glutaminase 2 (GLS2), glutamate dehydrogenase 1 (GLUD1), and glutamic-oxaloacetic transaminase 2 (GOT2), to support cell proliferation. Surprisingly, cell crisis is rescued only completely by supplementation with asparagine but minimally by supplementation with α-ketoglutarate, aspartate, or glutamate upon glutamine deprivation, implying an essential role of γ-nitrogen in glutamine and asparagine for cell proliferation. Specifically, glutamine and asparagine provide the critical γ-nitrogen for purine and pyrimidine biosynthesis, as knockdown of four rate-limiting enzymes in the pathways, including carbamoylphosphate synthetase 2 (CAD), phosphoribosyl pyrophosphate amidotransferase (PPAT), and phosphoribosyl pyrophosphate synthetases 1 and 2 (PRPS1 and PRPS2, respectively), suppresses cell proliferation. These findings indicate that glutamine and asparagine are shunted to the biosynthesis of nucleotides and nonessential amino acids from the tricarboxylic acid (TCA) cycle to support the anabolic proliferation of KSHV-transformed cells. Our results illustrate a novel mechanism by which an oncogenic virus hijacks a metabolic pathway for cell proliferation and imply potential therapeutic applications in specific types of cancer that depend on this pathway.

## IMPORTANCE

We have previously found that Kaposi’s sarcoma-associated herpesvirus (KSHV) can efficiently infect and transform primary mesenchymal stem cells; however, the metabolic pathways supporting the anabolic proliferation of KSHV-transformed cells remain unknown. Glutamine and asparagine are essential for supporting the growth, proliferation, and survival of some cancer cells. In this study, we have found that KSHV accelerates glutamine metabolism by upregulating numerous critical metabolic enzymes. Unlike most cancer cells that primarily utilize glutamine and asparagine to replenish the TCA cycle, KSHV-transformed cells depend on glutamine and asparagine for providing γ-nitrogen for purine and pyrimidine biosynthesis. We identified four rate-limiting enzymes in this pathway that are essential for the proliferation of KSHV-transformed cells. Our results demonstrate a novel mechanism by which an oncogenic virus hijacks a metabolic pathway for cell proliferation and imply potential therapeutic applications in specific types of cancer that depend on this pathway.

## INTRODUCTION

For many decades, most studies in cancer metabolism have been focusing on glucose following Otto Warburg’s pioneering work on aerobic glycolysis (1). The recent resurgence of interest in cancer metabolism has extended beyond glucose metabolism to include other nutrients such as glutamine because of the complexity of the cancer microenvironment. A complete picture of cancer metabolism should take into consideration the simultaneous contributions of multiple nutrients (2).

Glutamine, as the most abundant amino acid in the plasma and the major carrier of nitrogen, is used as a source of both energy and carbon and nitrogen backbones to support biomass accumulation and cellular homeostasis (3). As a result, glutamine is often exploited to support the anabolic growth of cancer cells. Indeed, most cancer cells consume and utilize more glutamine than other amino acids (3, 4). The reliance of cancer cells on glutamine metabolism has been observed in numerous tumorigenic contexts and is enhanced by the expression of proto-oncogenes c-myc and E2F, which control the expression of genes involved in glutamine metabolism (3–5). Consequently, compounds targeting glutamine metabolism are being actively pursued for anticancer therapy (3, 6–9), and ^18^F-labeled glutamine tracers are used for cancer diagnosis and prognosis in preclinical and early clinical studies (10).

Besides being directly involved in protein synthesis, the metabolic fates of glutamine can be roughly divided into reactions that use its γ-nitrogen and those that use either the α-nitrogen or the carbon skeleton (4). The γ-nitrogen amide group of glutamine is an indispensable donor of nitrogen for *de novo* synthesis of both nucleobases purine and pyrimidine and hexosamines such as glucosamine and galactosamine (3). The reactions in the second category use glutamate, which is converted from glutamine by glutaminase in mitochondria, as the substrate. Glutamate is converted to α-ketoglutarate (α-KG) to fuel the tricarboxylic acid (TCA) cycle through anaplerosis (3, 4). This reaction is performed by either glutamate dehydrogenase (GLUD1 and GLUD2 in humans) or several aminotransferases which transfer the α-nitrogen from glutamate to produce another amino acid and α-KG (3, 11). Hence, α-KG has been proposed as an essential metabolite for cell survival in glutamine-dependent cancer cells (12, 13). However, the full spectrum of glutamine-dependent tumors and the underlying mechanisms by which glutamine contributes to the anabolic proliferation of cancer cells remain an area of active investigation.

Asparagine is structurally similar to glutamine since both of them contain amide groups in their respective side chains. The importance of asparagine for tumor growth has been demonstrated in leukemia cells expressing a low level of asparagine synthetase (ASNS) (14). Unlike the other 19 common amino acids, the only reported use of asparagine in mammalian cells is in protein synthesis. However, results of two recent studies suggest a crucial regulatory role of asparagine in cancer cells, which is more than that of a mere substrate for protein synthesis (15, 16).

Kaposi’s sarcoma-associated herpesvirus (KSHV), one of the seven human oncogenic viruses, is causally associated with the development of Kaposi’s sarcoma (KS) and primary effusion lymphoma (PEL) (17). Despite intensive investigations, the mechanism underlying KSHV-induced malignant transformation remains unclear. Recent studies have shown that KSHV infection alone is sufficient to trigger cellular metabolic reprogramming (18–22). KSHV infection induces the Warburg effect in human endothelial cells and promotes lipogenesis in endothelial cells and PEL cells (18–20). KSHV-infected endothelial cells are glutamine addicted and require glutaminolysis for survival (21). Nevertheless, KSHV infection of primary human endothelial cells does not lead to cellular transformation.

We have recently reported that metabolic reprogramming is essential for KSHV-induced cellular transformation in a model of KSHV-induced cellular transformation of primary rat mesenchymal stem cells (MM cells) (23, 24). To our surprise, we have discovered that, in contrast to untransformed KSHV-infected endothelial cells (19, 22), KSHV suppresses aerobic glycolysis in the transformed cells. Moreover, KSHV-transformed cells (KMM cells) do not require glucose for proliferation and cellular transformation, and this metabolic reprogramming is essential for adaptation to glucose deprivation, which is one of the common stress conditions in the tumor microenvironment. Two major glucose transporters, GLUT1 and GLUT3, are downregulated in KS tumor cells in KS lesions, indicating the clinical relevance of these observations. In this study, we attempt to identify the nutrients that support the anabolic proliferation of KSHV-transformed cells and its underlying metabolic pathways. We have discovered that KSHV upregulates multiple enzymes to accelerate glutamine metabolism, providing the critical γ-nitrogen for the biosynthesis of nucleotides. We have also identified a novel function of asparagine in the biosynthesis of nucleotides, rather than it merely serving as an amino acid substrate for protein synthesis. These findings demonstrate a unique mechanism by which an oncogenic virus hijacks the metabolic pathways to support cellular transformation.

## RESULTS

### KSHV-transformed cells require glutamine for cell proliferation, survival, and formation of colonies in soft agar.

**FIG 1:**
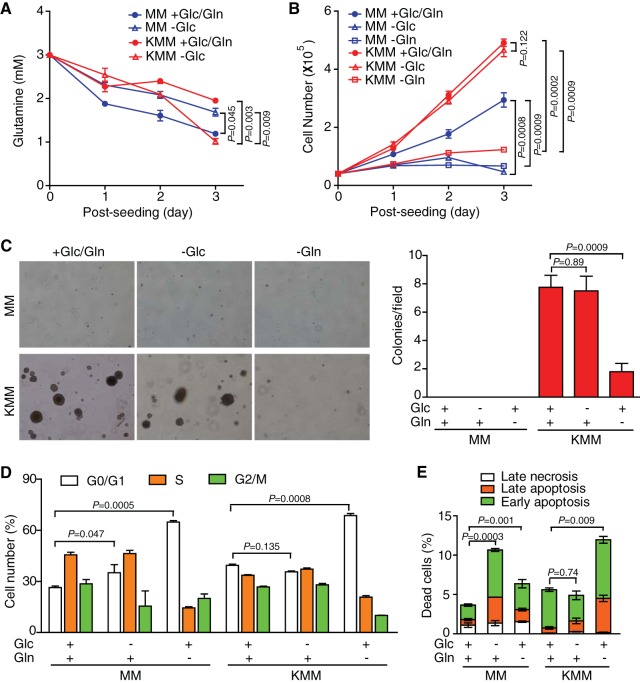
KSHV-transformed cells require glutamine for cell proliferation, survival, and formation of colonies in soft agar. (A) Glutamine levels in the media of untransformed (MM) and transformed (KMM) cells in the presence or absence of glucose. (B) KMM cells require glutamine but not glucose for proliferation. Cells seeded at 4 × 10^4^ cells/well in 24-well plates in complete medium containing 25 mM glucose and 4 mM glutamine overnight were replaced with glucose-free (-Glc), glutamine-free (-Gln), or complete (+Glc/Gln) medium, and cell numbers were counted daily. (C) KMM cells require glutamine but not glucose for the formation of colonies in soft agar. MM and KMM cells were plated in soft agar in complete, glucose-free, or glutamine-free medium for 14 days. Representative pictures captured at ×40 magnification are presented in the left panel. Colonies with diameters of >50 μm were counted, and colony numbers in each field are presented in the right panel. (D) Glutamine deprivation induces cell cycle arrest of both MM and KMM cells. Cell cycle distribution was analyzed by flow cytometry following 24-h glucose or glutamine deprivation. (E) Glutamine deprivation induces apoptosis of both MM and KMM cells. Apoptotic cells were detected by annexin V staining following 48 h of glucose or glutamine deprivation.

Our previous studies have shown that KSHV-transformed cells do not require glucose for proliferation and transformation (24), implying that they might utilize other nutrients to support their proliferation. To determine what nutrients might support the proliferation of KSHV-transformed cells, we first examined the consumption of glutamine, the second principal growth-supporting nutrient for most normal and cancer cells, by KSHV-transformed cells upon glucose deprivation. KSHV-transformed cells (KMM) consumed more glutamine than untransformed cells (MM) (Fig. 1A).

Next, we examined the dependence of transformed cells on glutamine. Upon glutamine deprivation, transformed cells completely arrested and failed to form colonies in soft agar (Fig. 1B and C). Consistent with results of our previous study (24), transformed cells did not depend on glucose for proliferation and formation of colonies in soft agar (Fig. 1B and C). The proliferation of untransformed cells was also sensitive to glutamine deprivation, as expected (Fig. 1B). Glutamine deprivation induced G_1_ arrest and increased the number of apoptotic cells in both untransformed and transformed cells (Fig. 1D and E). Collectively, these results indicate that KSHV-transformed cells require glutamine for cell proliferation, survival, and formation of colonies in soft agar.

**FIG 2:**
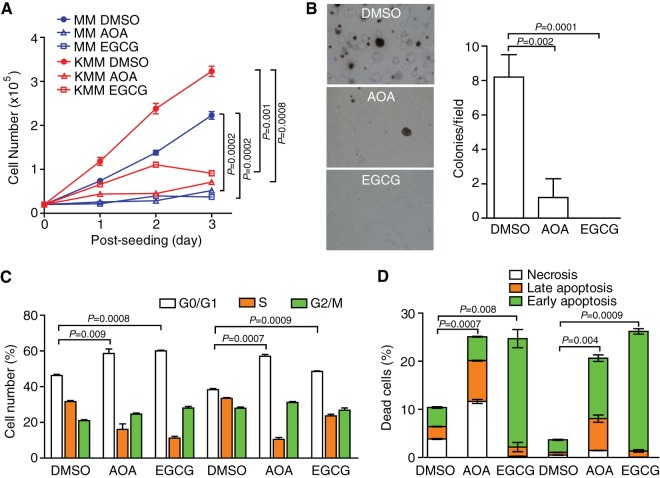
Inhibition of glutamine pathway with chemical inhibitors suppresses cell proliferation and cellular transformation of KSHV-transformed cells. (A) Epigallocatechin gallate (EGCG) and amino-oxyacetate (AOA) suppress cell proliferation of untransformed (MM) and KSHV-transformed (KMM) cells. MM and KMM cells were treated with GLUD1 inhibitor EGCG (100 μM) or transaminase inhibitor AOA (1 mM), and cell numbers were counted daily. (B) AOA and EGCG suppress colony formation of KMM cells in soft agar. Colony formation in soft agar was examined in the presence of 100 μM EGCG or 1 mM AOA. (C) Inhibition of glutamine pathway induces cell cycle arrest. Cell cycle of MM and KMM cells was analyzed following treatment of 100 μM EGCG or 1 mM AOA for 24 h. (D) Inhibition of glutamine pathway induces apoptosis. Apoptosis of MM and KMM cells was examined following treatment with 100 μM EGCG or 1 mM AOA for 48 h. DMSO, dimethyl sulfoxide.

Once imported into the cells, glutamine is converted to ammonium ion and glutamate by mitochondrial glutaminases. Glutamate can then be converted into α-KG to replenish the TCA cycle through two divergent pathways: by either glutamate dehydrogenase (GLUD1 in rat) or aminotransferases. We impaired these two divergent pathways using the inhibitors epigallocatechin gallate (EGCG) and amino-oxyacetate (AOA), respectively. EGCG is an inhibitor of GLUD1-mediated glutamate deamination, while AOA is a pan-inhibitor of transaminases. Both inhibitors significantly reduced cell proliferation rates in untransformed and transformed cells and reduced colony formation of transformed cells in soft agar (Fig. 2A and B). Consistent with these results, inhibition of glutamine metabolism induced cell cycle arrest and sensitized cells to apoptosis (Fig. 2C and D). Together, these results further confirm that glutamine is required for cell proliferation and cellular transformation of KSHV-transformed cells.

### GLS2, GLUD1, and GOT2 are upregulated and required for cell proliferation of KSHV-transformed cells.

**FIG 3:**
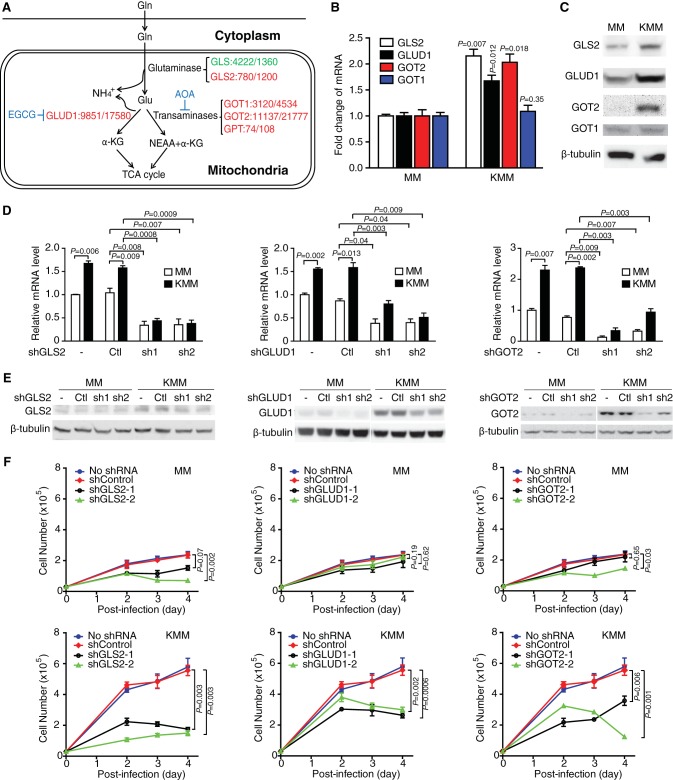
GLS2, GLUD1, and GOT2 are upregulated and required for cell proliferation of KSHV-transformed cells. (A) Changes of gene expression of key enzymes in the glutamine metabolism pathway following KSHV transformation. Ratios are untransformed (MM) versus transformed (KMM) cells. Genes downregulated and upregulated by KSHV are labeled in green and red, respectively. (B and C) Analysis of GLS2, GLUD1, GOT1, and GOT2 expression in MM and KMM cells by RT-qPCR (B) and Western blotting (C). (D) Examination of the efficiencies of shRNAs against GLS2, GLUD1, and GOT2 in untransformed (MM) and KSHV-transformed (KMM) cells by RT-qPCR. MM and KMM cells infected with lentiviruses harboring 2 different shRNAs (sh1 and sh2) for each gene or a scrambled control (Ctl) were lysed at day 3 postinfection and examined by RT-qPCR. β-Actin was used as an internal control. (E) Examination of the efficiencies of shRNAs against GLS2, GLUD1, and GOT2 in MM and KMM cells by Western blotting. Cells were infected as described in the legend for panel D. β-Tubulin was used as an internal control for loading. (F) Knockdown of GLS2, GLUD1, or GOT2 impairs the proliferation of KMM cells. MM and KMM cells infected with lentiviruses harboring 2 different shRNAs for GLS2 (shGLS2-1 and shGLS2-2), GLUD1 (shGLUD1-1 and shGLUD1-2), and GOT2 (shGOT2-1 and shGOT2-2) or a scrambled control (shControl) were counted at different time points after infection.

**FIG 4:**
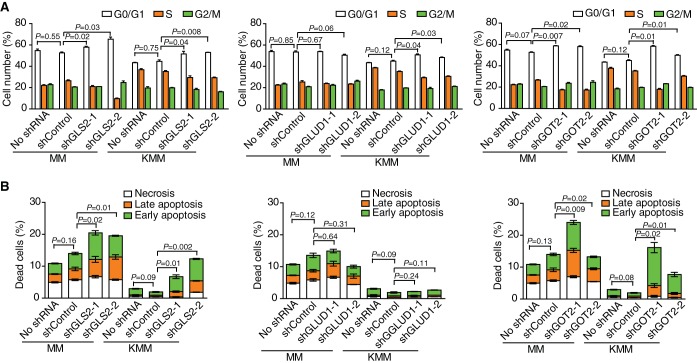
Knockdown of GLS2, GLUD1, or GOT2 induces cell cycle arrest and apoptosis. (A) Knockdown of GLS2, GLUD1, or GOT2 induces cell cycle arrest. Cell cycle distribution was analyzed by flow cytometry at 48 h after infection with lentiviruses as described in the legend to Fig. 3. (B) Knockdown of GLS2, GLUD1, or GOT2 induces apoptosis. Apoptotic cells were detected by annexin V staining at 72 h after infection with lentiviruses as described in the legend to Fig. 3.

To identify the mechanism of glutamine addiction of KSHV-transformed cells, we first performed high-throughput RNA sequencing and examined the changes of gene expression for key enzymes in the glutamine metabolic pathway. The expression levels of a panel of enzymes in the glutamine pathway, including glutaminase 2 (GLS2), GLUD1, and glutamic-oxaloacetic transaminases 1 and 2 (GOT1 and GOT2, respectively), were upregulated (Fig. 3A). Reverse transcription–real-time quantitative PCR (RT-qPCR) and Western blotting further confirmed the upregulation of GLS2, GLUD1, and GOT2 but not GOT1 at both mRNA and protein levels (Fig. 3B and C). To determine the roles of these enzymes in glutamine metabolism in KSHV-transformed cells, we designed two short hairpin RNAs (shRNAs) for each of these genes. Cells were transduced with lentiviruses expressing shRNAs against GLS2, GLUD1, or GOT2, which resulted in a significant reduction in both mRNA and protein levels of the respective genes (Fig. 3D and E). Significantly, knockdown of any of these genes inhibited cell proliferation of KSHV-transformed cells, reducing cell proliferation rates by 70% and 75% for GLS2, 50% and 55% for GLUD1, and 45% and 85% for GOT2. The GLS2 shRNAs reduced the proliferation rate of untransformed cells by 35% and 70%, but those of GLUD1 and GOT2 had minimal effects (Fig. 3F). Consistently, knockdown of GLS2, GLUD1, and GOT2 induced cell cycle arrest in transformed cells (Fig. 4A). Interestingly, knockdown of GLS2 and GOT2 but not GLUD1 induced cell death in transformed cells (Fig. 4B), which was in agreement with the strong inhibitory effects of these shRNAs on cell proliferation (Fig. 3F). These results indicate that GLS2 and GOT2 have critical roles for glutamine metabolism in KSHV-transformed cells.

### KSHV-transformed cells are fully rescued by asparagine but not α-KG upon glutamine deprivation.

**FIG 5:**
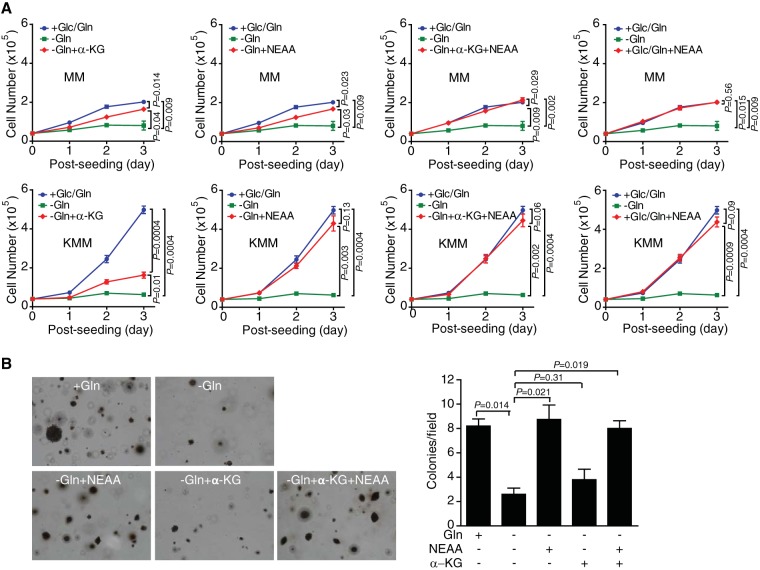
Nonessential amino acids (NEAA), but not TCA cycle replenishment, fully rescues KSHV-transformed cells from glutamine deprivation. (A) NEAA but not α-KG can fully rescue cell proliferation arrest of KSHV-transformed cells (KMM) from glutamine deprivation. Untransformed (MM) and KMM cells seeded at 4 × 10^4^ cells/well in 24-well plates in complete medium overnight were replaced with complete medium, glutamine-free medium, glutamine-free medium with α-KG, glutamine-free medium with NEAA, glutamine-free medium with α-KG and NEAA, or complete medium with NEAA, and cell numbers were counted daily. (B) NEAA but not α-KG can fully rescue colony formation of KMM cells in soft agar from glutamine deprivation. Formation of colonies in soft agar of cells treated with the indicated medium described in the legend to panel A.

Glutamate can be converted into α-KG through two different pathways: the GLUD pathway and the pathway of aminotransferases. The GLUD pathway generates ammonium, while the pathway of aminotransferases generates other nonessential amino acids (NEAA) such as serine, alanine, and aspartate, respectively, as by-products in these processes. To determine the roles of these pathways in glutamine metabolism in KSHV-transformed cells, we examined whether addition of permeable dimethyl α-KG as an exogenous carbon source or a mixture of nonessential amino acids, including alanine, aspartate, asparagine, glycine, proline, glutamate, and serine as nitrogen sources, can rescue the effect of glutamine deprivation. In untransformed cells, the cell proliferation rate was rescued by 75 to 82% by α-KG or NEAA alone and completely by a combination of α-KG and NEAA from arrest caused by glutamine deprivation. Surprisingly, in transformed cells, α-KG alone restored only 33% of the cell proliferation rate, whereas NEAA alone or the combination of α-KG and NEAA completely rescued cell proliferation from arrest caused by glutamine deprivation (Fig. 5A). Similar patterns were observed in a soft agar assay (Fig. 5B). Hence, the rescue effect of α-KG alone was significantly reduced in transformed cells compared to untransformed cells (33% versus 82%, respectively). Together, these results indicate that KSHV-transformed cells are more dependent on the NEAA converted from glutamine by aminotransferases, and KSHV might reprogram glutamine catabolism to meet the demand for nitrogen of rapidly dividing cells during cellular transformation.

**FIG 6:**
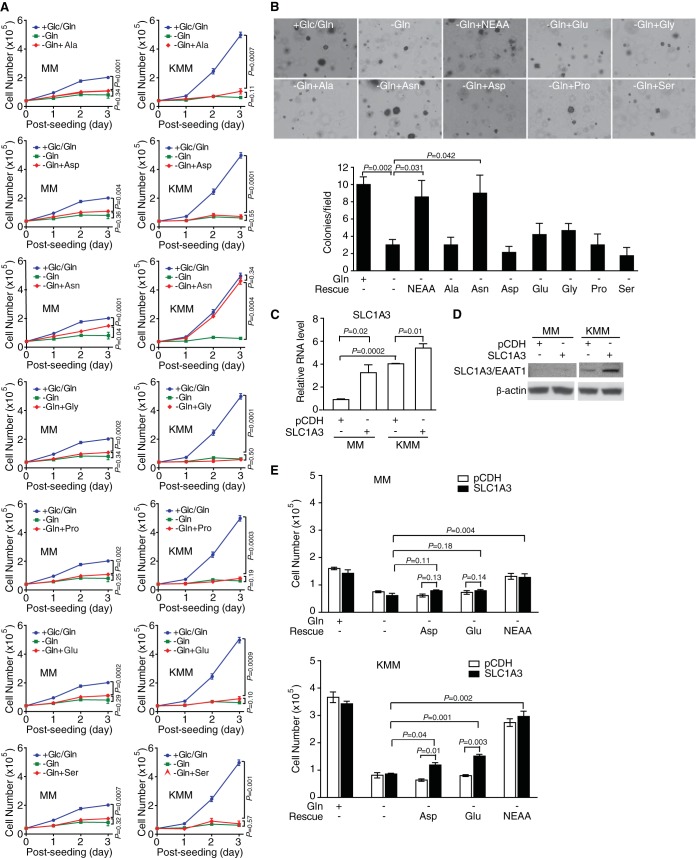
Asparagine alone, but not other nonessential amino acids (NEAA), is sufficient to rescue KSHV-transformed cells from glutamine deprivation. (A) Cell proliferation of untransformed (MM) and KSHV-transformed (KMM) cells in complete medium, glutamine-free medium, or glutamine-free medium with single NEAA alanine (Ala), aspartate (Asp), asparagine (Asn), glycine (Gly), proline (Pro), glutamate (Glu), or serine (Ser). (B) Formation of colonies in soft agar of cells treated with the indicated medium described in the legend to panel A. (C) Untransformed (MM) and KSHV-transformed (KMM) cells with stable expression of SLC1A3 were analyzed by RT-qPCR. β-Actin was used as an internal control. (D) MM and KMM cells with stable expression of SLC1A3 were analyzed by Western blotting. β-Actin was used as an internal control for loading. (E) Aspartate and glutamate can only partially support the proliferation of KMM cells under a glutamine-free condition after overexpression of glutamate aspartate transporter EAAT1 (SLC1A3). MM and KMM cells stably expressing SLC1A3 were cultured in glutamine-free medium containing 4 mM glutamate or 4 mM aspartate, and cell numbers were counted at day 3 after treatment.

To identify the component of NEAA that rescued KSHV-transformed cells upon glutamine deprivation, we tested the effect of each NEAA. We found that only asparagine could rescue cell proliferation arrest of KSHV-transformed cells upon glutamine deprivation (Fig. 6A). Under the same condition, asparagine only partially rescued cell proliferation arrest of untransformed cells (Fig. 6A). Consistent with these results, asparagine alone was also sufficient for rescuing the colony formation efficiency in soft agar of transformed cells upon glutamine deprivation (Fig. 6B). Interestingly, glutamate and aspartate were not able to rescue the cell proliferation arrest of transformed cells from glutamine deprivation, though they are structurally similar to glutamine and asparagine, respectively, except for the lack of an amide group. To ensure that the lack of rescue of glutamine deprivation by glutamate and aspartate was not due to the poor expression of the glutamate-aspartate transporter, we overexpressed the transporter SLC1A3 in untransformed and transformed cells (Fig. 6C and D) and then cultured them in glutamine-free medium, containing 4 mM glutamate or 4 mM aspartate. In the presence of glutamate or aspartate, overexpression of SLC1A3 only moderately prevented the proliferation arrest caused by glutamine deprivation of KSHV-transformed cells (Fig. 6E). Overexpression of SLC1A3 had no effect on the untransformed cells, which might be due to the relatively low expression of SLC1A3 in the stable cells (Fig. 6E). Taken together, we conclude that the amide group of glutamine or asparagine is a limiting factor for maintaining the proliferation of KSHV-transformed cells.

### GS is required for asparagine-dependent rescue of glutamine deprivation.

**FIG 7:**
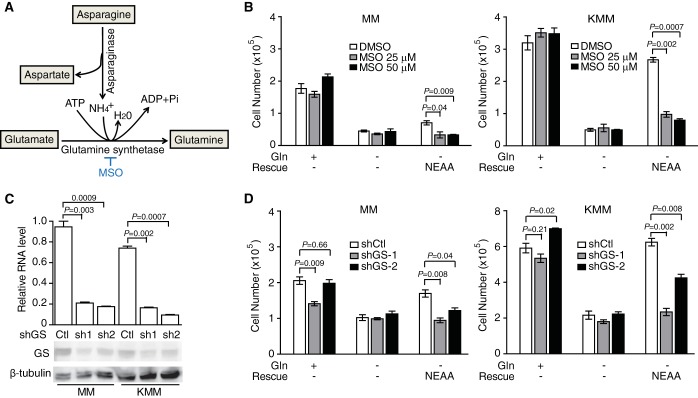
Chemical inhibition or knockdown of glutamine synthetase abolishes the rescue effect of NEAA in KSHV-transformed cells upon glutamine deprivation. (A) Schematic overview of reactions catalyzed by glutamine synthetase (GS). (B) GS inhibitor l-methionine sulfoximine (MSO) impairs the rescue effect of NEAA in KMM cells upon glutamine deprivation. Untransformed (MM) and KSHV-transformed (KMM) cells were grown with or without glutamine, NEAA, or MSO (25 μM or 50 μM) for 3 days, and cell numbers were counted. DMSO, dimethyl sulfoxide. (C) Knockdown efficiency of shRNAs against GS (shGS) in MM and KMM cells examined by RT-qPCR and Western blotting. (D) Knockdown of GS abolishes the rescue effect of NEAA in KMM cells upon glutamine deprivation. MM and KMM cells infected with lentiviruses harboring 2 different GS shRNAs (shGS-1 and shGS-2) or a scrambled control (shCtl) for 24 h were grown with or without glutamine and NEAA for 3 days, and cell numbers were counted.

The evidence above suggests that asparagine is required for the proliferation of KSHV-transformed cells upon glutamine deprivation. Asparagine is essential for some types of cancer cells, and l-asparaginase therapy is effective for the treatment of acute lymphoblastic leukemia lacking asparagine synthetase (14). Both asparagine and glutamine contain a γ-amide group and are thus structurally similar. However, except for directly supporting protein synthesis and forming aspartate and ammonia via asparaginase-catalyzed hydrolysis, asparagine has not been reported to be utilized in other synthetic pathways (25) and hence is considered metabolically essential and inert. We hypothesized that asparagine donates ammonia for glutamine synthesis (Fig. 7A). In order to investigate the mechanism, we determined whether asparagine was involved in glutamine synthesis in KSHV-transformed cells by applying a glutamine synthetase (GS) inhibitor, l-methionine sulfoximine (MSO). MSO abolished the rescue effect of asparagine on KSHV-transformed cells upon glutamine deprivation (Fig. 7B). Accordingly, knockdown of GS with shRNAs had similar results (Fig. 7C and D). Collectively, our results suggest that asparagine rescues KSHV-transformed cells by supplying γ-nitrogen from the amide group for glutamine synthesis upon glutamine deprivation.

### Supplementation with nucleosides, glutamate, and α-KG enables anabolic proliferation of KSHV-transformed cells upon glutamine deprivation.

**FIG 8:**
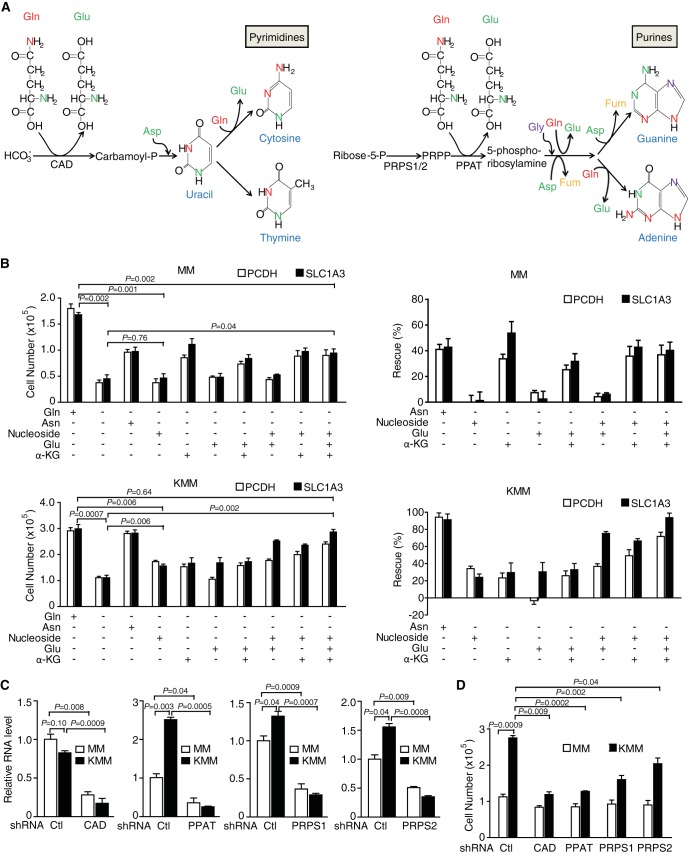
A combination of nucleosides, glutamate, and α-KG fully rescues KSHV-transformed cells upon glutamine deprivation. (A) Schematic illustrations of sources of nitrogen atoms of nucleotides at different positions from glutamine and glutamate and the related enzymes of nucleotide biosynthesis. PRPP, phosphoribosyl pyrophosphate; PRPS1/2, phosphoribosyl pyrophosphate synthetase 1/2; CAD, carbamoylphosphate synthetase 2; Gln, glutamine; Glu, glutamate; Gly, glycine; Asp, aspartate; Fum, fumarate. γ-Nitrogen atoms and α-nitrogen atoms are labeled in red and green, respectively. (B) Untransformed (MM) and KSHV-transformed (KMM) cells with stable expression of SLC1A3 were cultured in medium with or without glutamine and with and without addition of different combinations of asparagine, nucleoside, glutamate, and α-KG for 72 h, and the cell numbers were counted. For the calculation of percentages of rescue, the difference of cell numbers between cells cultured in the complete medium and those cultured in glutamine-free medium was set as 100%. (C) Analysis of knockdown efficiency of shRNAs against carbamoylphosphate synthetase 2 (shCAD), phosphoribosyl pyrophosphate amidotransferase (shPPAT), and phosphoribosyl pyrophosphate synthetases 1 and 2 (shPRPS1 and shPRPS2, respectively) in MM and KMM cells by RT-qPCR. (D) Knockdown of CAD, PPAT, or PRPS1/2 impairs cell proliferation of KMM cells. MM and KMM cells were infected with lentiviruses harboring shRNAs of the respective gene or a scrambled control (Ctl) for 72 h, and cell numbers were counted.

Our results have so far shown that the amide group of glutamine, an indispensable donor of reduced nitrogen for the biosynthesis of purine and pyrimidine bases (Fig. 8A), is a rate-limiting source for the anabolic proliferation of KSHV-transformed cells. To explore the role of nucleotides in supporting the proliferation of KSHV-transformed cells, we determined whether the addition of permeable nucleosides could counteract the effect of glutamine deprivation. Indeed, nucleosides partially rescued transformed but not untransformed cells upon glutamine deprivation (Fig. 8B). Moreover, the combination of nucleosides, glutamate, and α-KG fully rescued the arrest of cell proliferation caused by glutamine deprivation (Fig. 8B). These results indicate that both a nitrogen source for the biosynthesis of nucleotides and nonessential amino acids and a carbon source for the TCA cycle are required for the anabolic proliferation of KSHV-transformed cells.

To further confirm the essential role of glutamine-dependent nucleotide biosynthesis in supporting the proliferation of KSHV-transformed cells, we performed knockdown of three rate-limiting enzymes in the *de novo* purine biosynthesis pathway, phosphoribosyl pyrophosphate amidotransferase (PPAT) and phosphoribosyl pyrophosphate synthetases 1 and 2 (PRPS1/2), and one rate-limiting enzyme in the pyrimidine biosynthesis pathway, carbamoylphosphate synthetase 2 (CAD) (Fig. 8C). Knockdown of CAD, PPAT, or PRPS1/2 dramatically impaired the proliferation of transformed cells but not untransformed cells (Fig. 8D). Therefore, the biosynthesis of nucleotides is required for the anabolic proliferation of KSHV-transformed cells.

Taken together, these results indicate that KSHV has reprogrammed the glutamine metabolic pathways to endow them with the potential for biosynthesis of nucleotides and nonessential amino acids, which are essential for the anabolic proliferation and cellular transformation of KSHV-transformed cells.

## DISCUSSION

We have previously shown that the oncogenic virus KSHV suppresses aerobic glycolysis (24). This reprogrammed metabolic program promotes the survival and cellular transformation of KSHV-infected cells, particularly under conditions deprived of nutrients such as glucose, which could be in part due to the increased SIRT1 and AMP-activated protein kinase (AMPK) activities (26, 27). Furthermore, low glucose metabolism promotes KSHV latency as a result of increasing activities of SIRT1 and AMPK, which suppress viral lytic replication (28, 29), and decreasing reactive oxygen species, which enhance viral lytic replication (30, 31). In the current study, we have shown that by upregulating the expression of a panel of enzymes including GLS2, GLUD1, and GOT2, KSHV-transformed cells have an increased level of glutamine metabolism. Glutamine provides not only a carbon skeleton for the TCA cycle but also a nitrogen source for the biosynthesis of nucleotides and nonessential amino acids to meet the high demand of anabolic proliferation. Whereas untransformed cells depend on glutamine for a carbon source, KSHV-transformed cells depend on glutamine for both the nitrogen and carbon sources for cell proliferation and transformation. Addition of asparagine but not aspartate or glutamate alone is sufficient to completely rescue the proliferation arrest caused by glutamine depletion, pointing to a critical role of γ-nitrogen of glutamine and asparagine for the anabolic proliferation of KSHV-transformed cells. Taken together, these results reveal a novel mechanism by which an oncogenic virus rewires the metabolic pathways to support the anabolic proliferation of cellular tumorigenic properties by redistributing the use of glutamine.

Glucose has a central role in supporting the anabolic proliferation of many types of cancer (2). However, up to 30% of cancers do not have active consumption of glucose, and therapies aiming to inhibit glucose utilization are not always efficacious because numerous aggressive tumors have evolved to counter glucose deprivation in the tumor microenvironment by reprogramming the metabolic pathways (32). The fact that KSHV-transformed cells do not require glucose for proliferation and cellular transformation implies that they might utilize alternative nutrients to support their proliferation. Indeed, glucose withdrawal increases glutamine consumption of KSHV-transformed cells (Fig. 1A). Furthermore, KSHV-transformed cells depend on glutamine but not glucose for cell proliferation and cellular transformation (Fig. 1B and C). These findings are consistent with results from another study showing that cancer cells with protein kinase C (PKC) deficiency have the plasticity to rewire their metabolic pathways so as to utilize glutamine in the absence of glucose (33). It is important to note that, under normal conditions, KSHV-transformed cells consume less glucose and glutamine than the untransformed cells despite their higher proliferation rate (24), indicating that they have likely optimized the metabolic pathways for the efficient utilization of both nutrients.

**FIG 9:**
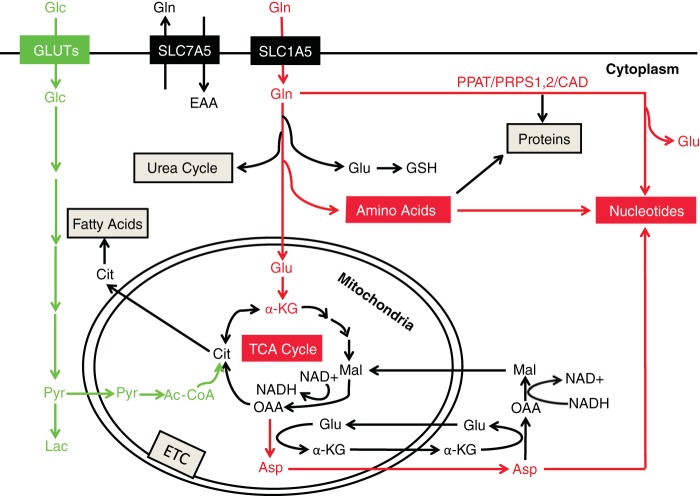
Schematic illustration of upsurge of glutamine metabolic pathways by KSHV. Glutamine and glucose metabolic pathways regulated by KSHV during cellular transformation are marked in red and green, respectively. GSH, glutathione; OAA, oxaloacetate; ETC, electron transport chain.

Glutamine, as a second principal nutrient, contributes not only carbon but also nitrogen for the *de novo* biosynthesis of diverse nitrogen-containing compounds, including nucleotides and nonessential amino acids (4). We have shown that KSHV-transformed cells depend on glutamine for their growth, proliferation, and survival, which is similar to the observations in many other types of cancer cells (3). In contrast to other types of cancer cells, which consume large amounts of glutamine to fuel the anaplerosis of the TCA cycle (12, 13), we have found that NEAA or asparagine but not dimethyl α-KG can restore the proliferation of KSHV-transformed cells upon glutamine deprivation (Fig. 3A and 4A), indicating an essential role of glutamine in supplying the nitrogen source. In fact, the amide group of glutamine is an essential donor of nitrogen for the biosynthesis of purine and pyrimidine bases (5). In addition, the assembly of both pyrimidine and purine utilizes aspartate, which is derived from the transamination of oxaloacetate and glutamic acid from the TCA cycle, both of which are catabolites of glutamine. Thus, glutamine is a key structural building block in the biosynthesis of nucleotides (3). Accordingly, the level of glutamine is a rate-limiting factor for cell cycle progression (34), and we have shown that glutamine deprivation leads to cell cycle arrest in G_1_ phase in KSHV-transformed cells (Fig. 1D). It was reported that glutamine-deprived cancer cells undergo cell cycle arrest, which could be rescued by exogenous nucleotides but not intermediates of the TCA cycle such as oxaloacetate (35, 36). Moreover, α-nitrogen from glutamine can be transferred by aminotransferases to produce other NEAA, such as serine, alanine, and aspartate, which are required for other metabolic pathways. For example, besides involvement in nucleotide synthesis and the aspartate-malate shuttle (Fig. 9), aspartate has recently been shown to be critical for oxidative phosphorylation (37, 38). Interestingly, although a key characteristic of glutamine is that it provides the nitrogen source for the biosynthesis of nucleotides and NEAA in KSHV-transformed cells, it also provides a carbon skeleton for the TCA cycle (Fig. 8B). These results could be explained by the fact that KSHV-transformed cells do not require glucose-derived carbon for proliferation and therefore depend on glutamine to fulfill the carbon demand of the TCA cycle (Fig. 1A). Importantly, although glutamate could supply α-KG for the TCA cycle through glutamate dehydrogenase, additional α-KG is required for fully rescuing the proliferation of KSHV-transformed cells from glutamine depletion (Fig. 8B). It is likely that the TCA cycle provides intermediates for the synthesis of amino acids and lipids (Fig. 9).

Although several amino acids can be derived from glutamine through its catabolism to glutamate, asparagine can be derived from glutamine only via asparagine synthetase (ASNS). Asparagine is essential for the proliferation of numerous types of cancer, and extracellular asparaginase therapy has been effectively used for treating low-ASNS-expressing leukemia (14). While cancer cells have a high demand for asparagine, most of them are self-sufficient and synthesize asparagine *de novo* via ASNS. Genetic silencing of ASNS in sarcoma cells combined with asparaginase therapy in plasma was shown to blunt tumor growth *in vivo* (39). Asparagine and glutamine are structurally similar since they both contain amide groups in their respective side chains. However, most normal mammalian tissues are unable to catabolize asparagine, with the exception of liver and kidney (25). The only currently known function of asparagine is to be used as a substrate for protein synthesis. Despite our limited knowledge of asparagine functions, results of two recent papers suggest that it has functions other than as a mere substrate for protein synthesis. In one study, it was shown that asparagine suppressed cell death in cells that depended on glutamine for survival (16). However, exactly how asparagine supported cell survival and adaptation to glutamine depletion was not elucidated. In a second study, it was shown that asparagine promoted the proliferation of liposarcoma and breast cancer cell lines by serving as an amino acid exchange factor (15). Results of both studies suggest a crucial regulatory role of asparagine in cancer cells under conditions of glutamine deprivation. Consistent with these studies, our results have shown that among those seven NEAA, asparagine is the only one that can fully rescue the proliferation of KSHV-transformed cells from glutamine deprivation (Fig. 6A). Furthermore, aspartate and glutamate can only partially rescue KSHV-transformed cells upon glutamine deprivation after overexpressing their transporter EAAT1 (excitatory amino acid transporter 1) (Fig. 6E). It is worth mentioning that Dulbecco’s modified Eagle’s medium (DMEM), which was used to culture the untransformed and transformed cells, contains 0.03 g/liter glycine and 0.042 g/liter serine without any asparagine. Moreover, we have observed that three enzymes, ASPG (human asparaginase enzyme), ASRGL1 (asparaginase-like 1), and AGA (aspartylglucosaminidase), which can catalyze the hydrolysis of asparagine into aspartate and ammonia, are upregulated in KSHV-transformed cells compared to untransformed cells (data not shown), indicating that KSHV might optimize the metabolic pathways for the efficient utilization of asparagine. Taken together, these results indicate that the amide group from asparagine is indispensable for maintaining the proliferation of transformed cells, and the exchange role of asparagine is unlikely the main mechanism of its rescuing effect, since addition of other amino acids cannot achieve the same effect as that of asparagine.

Our results indicate that asparagine is involved in glutamine synthesis in KSHV-transformed cells upon glutamine deprivation (Fig. 7). Previous studies have shown that glutamine can be synthesized from glutamate and free ammonia through GS (40, 41). In fact, GS promotes biosynthesis of asparagine and nucleotides and is overexpressed in some types of cancer (42). Nevertheless, a combination of NH_4_Cl and glutamate was not able to rescue KSHV-transformed cells from glutamine deprivation even after overexpression of SLC1A3 (data not shown). Hence, we have concluded that asparagine is the only amino acid that can supply the nitrogen source in this reaction.

We have shown that the expression levels of multiple enzymes in the glutamine metabolic pathway, including GLS2, GLUD1, and GOT2, are upregulated in KSHV-transformed cells. This is the first time that these enzymes are shown to be regulated by an oncogenic virus. Unlike GLS, which is active in several types of cancer (3) and is indirectly promoted by c-myc through repressing the expression of miR-23a/b (43), the role of GLS2 in cancer seems more complex. GLS2 has been shown to have tumor-suppressive activities and can be induced by p53 and its related proteins p63 and p73 (44–47). However, in neuroblastoma, GLS2 is a critical downstream target of N-myc (48), suggesting that the role of GLS2 in cancer is context dependent. The mitochondrial GOT2 isoform is considered to mainly contribute to the transfer of nitrogen from glutamate to oxaloacetate for producing aspartate and α-KG, whereas the cytoplasmic GOT1 isoform is shown to catalyze the reversible transfer of an amino group between aspartate and glutamate (37). GLUD catalyzes the deamination of glutamate to produce α-KG and release ammonium in cancer (49). MYC upregulates both GLUD and aminotransferases (50). However, oncogenic mutant KRAS increases GOT1 and decreases GLUD mRNA levels (11). Numerous studies have reported that KSHV regulates the c-myc and KRAS pathways (17). Further work is warranted to identify the KSHV genes that regulate the glutamine pathway during cellular transformation.

The findings that KSHV-transformed cells are addicted to glutamine are similar to those reported in untransformed KSHV-infected endothelial cells (21). However, KSHV-infected endothelial cells require glutaminolysis, which supplies the carbon backbone of glutamine to the TCA cycle for survival, whereas KSHV-transformed cells are more dependent on glutamine as a nitrogen source. Such differences could be due to the fact that KSHV induces the Warburg effect in untransformed cells (19, 22) but suppresses aerobic glycolysis in transformed cells (24). Whether such variations are due to the state of metabolism in different cell types or the state of cellular transformation remains to be determined.

In summary, KSHV accelerates glutamine metabolism by hijacking multiple enzymes to supply the essential transfer of γ-nitrogen for nucleotide biosynthesis in cancer cells. Our results have identified a novel function of asparagine, which is to provide γ-nitrogen for purine and pyrimidine biosynthesis rather than merely serve as an amino acid substrate for protein synthesis. These findings illustrate the essential role of metabolic reprogramming in cellular transformation and tumorigenesis, which could be exploited for therapeutic application.

## MATERIALS AND METHODS

### Cell culture and reagents.

Rat primary embryonic metanephric mesenchymal stem cells (MM), KSHV-transformed MM cells (KMM), and 293T cells were maintained in Dulbecco’s modified Eagle’s medium (DMEM) containing 25 mM glucose, 4 mM l-glutamine, and 2 mM sodium pyruvate supplemented with 10% fetal bovine serum (FBS; Sigma-Aldrich, St. Louis, Mo), 100 μg/ml penicillin, and 100 μg/ml streptomycin. For glutamine deprivation, cells were cultured in DMEM without glutamine containing 25 mM glucose and 2 mM sodium pyruvate supplemented with 10% FBS. For glucose deprivation, cells were cultured in DMEM without glucose containing 4 mM l-glutamine and 2 mM sodium pyruvate supplemented with 10% FBS. *O*-(Carboxymethyl)hydroxylamine hemihydrochloride, (AOA; catalog no. C13408), epigallocatechin gallate (EGCG; catalog no. E4143), l-methionine sulfoximine (MSO; catalog no. M5379), dimethyl 2-oxoglutarate (dimethyl α-KG; catalog no. 349631), and amino acids alanine (Ala), aspartate (Asp), asparagine (Asn), glycine (Gly), proline (Pro), glutamate (Glu), and serine (Ser) were purchased from Sigma-Aldrich. A 100× solution of nonessential amino acids (NEAA) (catalog no. SH30238.01) was obtained from HyClone (Logan, UT). A 100× nucleoside solution (catalog no. CMES008D) was obtained from EMD Millipore (Bedford, MA).

### Colony formation in soft agar.

A soft agar assay was performed as previously described (24). Briefly, a total of 2 × 10^4^ cells suspended in 1 ml of 0.3% top agar (catalog no. A5431; Sigma-Aldrich) was plated onto one well of 0.5% base agar in 6-well plates and maintained for 2 to 3 weeks. Colonies with a diameter of >50 μm were counted and photographed at ×40 magnification using a microscope.

### Cell cycle analysis and apoptosis assay.

The cell cycle was analyzed by propidium iodide (PI) staining at the indicated time points as previously described (24). Apoptotic cells were detected by staining with the fixable viability dye eFluor 660 (catalog no. 650864; eBioscience, San Diego, CA) and a phycoerythrin (PE)-Cy7 annexin V apoptosis detection set (catalog no. 88810374; eBioscience) according to the instructions of the manufacturer. Flow cytometry was performed in a FACSCanto system (BD Biosciences, San Jose, CA), and analysis was performed with FlowJo (FlowJo, LLC, Ashland, OR).

### Measurements of glutamine concentration.

Cells seeded in 24-well plates for 24 h were replaced with new medium, and assays were carried out at the indicated time points in normal medium or in glucose-free medium (glucose starvation). Glutamine concentrations were measured in the culture medium using the glutamine/glutamate determination kit (catalog no. GLN-1; Sigma-Aldrich) according to the manufacturer’s instructions.

### Lentiviral expression of SLC1A3.

The coding sequence of rat SLC1A3 (GenBank accession number NM_019225.2) was cloned into the XbaI/EcoRI sites of pCDHCMV-MCS-EF1-puro lentiviral vector (System Biosciences, Palo Alto, CA) by PCR amplification to generate expression vectors named pCDH-puro-SLC1A3. The primer sequences used for cloning are listed in Table 1. The construct was confirmed by direct DNA sequencing. Lentivirus-containing supernatants were collected and used for transduction as previously described (24). MM-vector, KMM-vector, MM-SLC1A3, and KMM-SLC1A3 cells were selected with 2 μg/ml puromycin after transduction.

**TABLE 1:** PCR and cloning primers

Purpose and name	Primer sequence
RT-qPCR	
GLS2	GAGGCTTGCAAAGTGAATCC (sense)
	AGTCTCAGCTGCTACCTCAGC (antisense)
GLUD1	TATCCGGTACAGCACTGACG (sense)
	GCTCCATGGTGAATCTTCGT (antisense)
GOT2	CTTACGTGCTCCCCAGTGTT (sense)
	CGGAAATGGTCTGCACAGTT (antisense)
GOT1	GACAACAGCCCAGCTCTCA (sense)
	GGCAGAAAACACGCCATTAT (antisense)
SLC1A3	TGACAAAAAGCAACGGAGAA (sense)
	GCTGACGGTGAGTAGCACAA (antisense)
GS	TCTGGACTGTGACCCCAAGT (sense)
	ACTTCGCAGAACACCAGCTT (antisense)
CAD	CCACTGGAGTGCCACCTG (sense)
	GCTGAAGGCTCTGTCCCTTT (antisense)
PPAT	AATAGCTGTGGCCCATAACG (sense)
	CTTGTTCCTGAGGAGGGGTA (antisense)
PRPS1	AATTGGGGAGAGTGTTCGTG (sense)
	TAAGGGAAGCATGGGATGAC (antisense)
PRPS2	CTCTTCAGCGGCAGCTCT (sense)
	CAGCCGCTCTGGATGATATAG (antisense)
β-Actin	GCAGGAGTACGATGAGTCCG (sense)
	ACGCAGCTCAGTAACAGTCC (antisense)

Cloning (restriction enzyme sites are underlined)	
SLC1A3	AGTTCTAGAATGACAAAAAGCAACGGAGAA (sense)
	AGTGAATTCCTACATCTTGGTTTCGCTGTC (antisense)
shGLS2-1	CCGGGGCCATGTGGATCGTATATTTCTCGAGAAATATACGATCCACATGGCCTTTTTG (sense)
	AATTCAAAAAGGCCATGTGGATCGTATATTTCTCGAGAAATATACGATCCACATGGCC (antisense)
shGLS2-2	CCGGGGGATCGGAATTACGCCATTGCTCGAGCAATGGCGTAATTCCGATCCCTTTTTG (sense)
	AATTCAAAAAGGGATCGGAATTACGCCATTGCTCGAGCAATGGCGTAATTCCGATCCC (antisense)
shGLUD1-1	CCGGGCGTTAAGATCAATCCCAAGACTCGAGTCTTGGGATTGATCTTAACGCTTTTTG (sense)
	AATTCAAAAAGCGTTAAGATCAATCCCAAGACTCGAGTCTTGGGATTGATCTTAACGC (antisense)
shGLUD1-2	CCGGGCTTGGCGATAAGACGTTTGTCTCGAGACAAACGTCTTATCGCCAAGCTTTTTG (sense)
	AATTCAAAAAGCTTGGCGATAAGACGTTTGTCTCGAGACAAACGTCTTATCGCCAAGC (antisense)
shGOT2-1	CCGGGGTGGACCCATGTTGAAATGGCTCGAGCCATTTCAACATGGGTCCACCTTTTTG (sense)
	AATTCAAAAAGGTGGACCCATGTTGAAATGGCTCGAGCCATTTCAACATGGGTCCACC (antisense)
shGOT2-2	CCGGGCGAGAACAGCGAAGTGTTGACTCGAGTCAACACTTCGCTGTTCTCGCTTTTTG (sense)
	AATTCAAAAAGCGAGAACAGCGAAGTGTTGACTCGAGTCAACACTTCGCTGTTCTCGC (antisense)
shGS-1	CCGGGCATCAAGCAGATGTACATGACTCGAGTCATGTACATCTGCTTGATGCTTTTTG (sense)
	AATTCAAAAAGCATCAAGCAGATGTACATGACTCGAGTCATGTACATCTGCTTGATGC (antisense)
shGS-2	CCGGGCGAAGTATTCAAGTATAACCCTCGAGGGTTATACTTGAATACTTCGCTTTTTG (sense)
	AATTCAAAAAGCGAAGTATTCAAGTATAACCCTCGAGGGTTATACTTGAATACTTCGC (antisense)
shCAD	CCGGGGGCTCCTCTATTTACCAACGCTCGAGCGTTGGTAAATAGAGGAGCCCTTTTTG (sense)
	AATTCAAAAAGGGCTCCTCTATTTACCAACGCTCGAGCGTTGGTAAATAGAGGAGCCC (antisense)
shPPAT	CCGGGGTATTGGGCTTTCCACATCCCTCGAGGGATGTGGAAAGCCCAATACCTTTTTG (sense)
	AATTCAAAAAGGTATTGGGCTTTCCACATCCCTCGAGGGATGTGGAAAGCCCAATACC (antisense)
shPRPS1	CCGGGCGGTGAGATAAATGACAATCCTCGAGGATTGTCATTTATCTCACCGCTTTTTG (sense)
	AATTCAAAAAGCGGTGAGATAAATGACAATCCTCGAGGATTGTCATTTATCTCACCGC (antisense)
shPRPS2	CCGGGCATCCTCATCCAGGGTAACACTCGAGTGTTACCCTGGATGAGGATGCTTTTTG (sense)
	AATTCAAAAAGCATCCTCATCCAGGGTAACACTCGAGTGTTACCCTGGATGAGGATGC (antisense)
shControl	CCGGTTGTACTACACAAAAGTACTGCTCGAGCAGTACTTTTGTGTAGTACAATTTTTG (sense)
	AATTCAAAAATTGTACTACACAAAAGTACTGCTCGAGCAGTACTTTTGTGTAGTACAA (antisense)

### Lentiviral shRNA knockdown.

To generate shRNA vectors, oligonucleotides of sense and antisense shRNA sequences targeting rat GLS2, GOT2, GLUD1, GS, CAD, PPAT, PRPS1, or PRPS2, together with the loop sequence (5′-CTCGAG) and the flanking AgeI and EcoRI sites, were designed. The oligonucleotide sequences used for cloning are listed in Table 1. Two complementary sequences were synthesized chemically, annealed, and ligated into the AgeI and EcoRI sites of linearized pLKO.1 lentiviral vector (Addgene, Cambridge, MA). Lentivirus-containing supernatants were collected, and transduction was performed as previously described (24). Cells transduced with the lentivirus particles were examined for knockdown efficiency at day 3 posttransduction.

### Reverse transcription–real-time quantitative PCR (RT-qPCR).

Total RNA was isolated with TRI reagent (catalog no. T9424) according to the instructions of the manufacturer (Sigma). Reverse transcription was performed with total RNA using the Maxima H Minus first-strand cDNA synthesis kit (catalog no. K1652; Thermo Fisher Scientific, Waltham, MA). Quantitative PCR (qPCR) analysis was performed on an Eppendorf RealPlex instrument using Kapa SYBR fast qPCR kits (catalog no. KK4602; Kapa Biosystems, Wilmington, MA). The relative expression levels of target genes were normalized to the expression levels of the internal control genes yielding cycle threshold (2^−ΔΔ*CT*^) values. All reactions were run in triplicates. The *C*_*T*_ values should not differ more than 0.5 among triplicates. The primer sequences used for cloning are listed in Table 1.

### Western blot analysis.

Total cell lysates were separated in SDS-polyacrylamide gels, electrophoretically transferred to nitrocellulose membranes (GE Healthcare, Piscataway, NJ). The membranes were incubated sequentially with primary and secondary antibodies. The signal was developed using Luminiata Crescendo Western horseradish peroxidase (HRP) substrate (catalog no. WBLUR0500; EMD Millipore, Billerica, MA). The antibodies used for Western blotting include rabbit monoclonal antibodies (MAbs) for GLUD1 (catalog no. 12793; Cell Signaling Technology, Inc., Danvers, MA), GOT1 (catalog no. ab170950; Abcam, Inc., Cambridge, MA), and EAAT1 (SLC1A3) (catalog no. 5684; Cell Signaling Technology, Inc.); rabbit polyclonal antibodies against GLS2 (catalog no. ab113509; Abcam, Inc.) and GS (catalog no. G2781; Sigma); and mouse MAbs for GOT2 (catalog no. ab93928; Abcam, Inc.), β-actin (catalog no. sc8432; Santa Cruz Biotechnology, Santa Cruz, CA), and β-tubulin (7B9, Sigma).

### Statistics.

Data were expressed as means ± standard errors of the means (SEMs) from at least three independent experiments, each with three repeats unless stated otherwise. The differences between groups were analyzed using Student’s *t* test when two groups were compared and one-way analysis of variance (ANOVA) when more than two groups were compared, unless otherwise noted. Statistical tests were two sided. A *P* value of <0.05 was considered statistically significant. All analyses were performed using the GraphPad Prism program (GraphPad Software, Inc., San Diego, CA).
